# Pilot study of ^18^F-FAPI-RGD PET/CT for the diagnosis of connective tissue disease associated interstitial lung disease

**DOI:** 10.1186/s12931-025-03367-7

**Published:** 2025-11-15

**Authors:** Hao Liu, Xing He, Hui Fang, Nan Liu, Liqing Yang, Caiyu Jiang, Wubin Long, Wei Zhang, Lu Guo

**Affiliations:** 1 Department of Nuclear Medicine, Sichuan Provincial People’s Hospital, School of Medicine, University of Electronic Science and Technology of China, Chengdu, Sichuan China; 2 Department of Pulmonary and Critical Care Medicine, Sichuan Provincial People’s Hospital, School of Medicine, University of Electronic Science and Technology of China, Chengdu, Sichuan China; 3 Department of Rheumatology and Immunology, Sichuan Provincial People’s Hospital, School of Medicine, University of Electronic Science and Technology of China, Sichuan Chengdu, China; 4 Department of Pulmonary and Critical Care Medicine, West China Hospital, Sichuan University, Chengdu, Sichuan China

**Keywords:** PET, ^18^F-FAPI-RGD, ^18^F-LNC1007, Connective tissue disease, Interstitial lung disease

## Abstract

**Background:**

^18^F-FAPI-RGD PET/CT is a dual-target tracer imaging technique that holds promise for identifying pulmonary lesions in connective tissue disease-associated interstitial lung disease (CTD-ILD) patients. Currently, clinical studies investigating ^18^F-FAPI-RGD PET/CT for CTD-ILD diagnosis are lacking.

**Methods:**

In this prospective study, CTD-ILD patients from the multidisciplinary clinic of Sichuan Provincial People’s Hospital were enrolled. General clinical data and laboratory parameters were collected. All participants underwent multidisciplinary ILD diagnostic evaluation and completed ^18^F-FAPI-RGD PET/CT examinations to assess its clinical value in CTD-ILD diagnosis.

**Results:**

The study ultimately included 52 patients (35 CTD-ILD, 17 CTD). CTD-ILD patients showed significantly elevated serum IL-6 and KL-6 levels compared to CTD patients (*P* < 0.05). ^18^F-FAPI-RGD PET/CT imaging revealed significantly higher bilateral lung SUVmax (*P* < 0.001) and lower spleen SUVmax (*P* < 0.05) in CTD-ILD patients. Correlation analysis demonstrated significant positive association between bilateral lung SUVmax and CTD-ILD (*r* = 0.62, *P* < 0.001). Multivariate logistic regression adjusted for age, KL-6, and DLCO% identified bilateral lung SUVmax (per 10-unit increase) as an independent predictor of CTD-ILD (OR = 1.459, 95% CI= [1.031–2.065], *P* = 0.033). Bilateral lung SUVmax exhibited excellent predictive value for ILD (AUC = 0.94, 95% CI= [0.88-1.00], *P* < 0.001). Multiple linear regression showed KL-6 (β = 0.001, 95%CI [< 0.001, 0.002], *P* < 0.001) significantly positively influenced bilateral lung SUVmax, while DLCO% (β=-0.031 95%CI [-0.043, -0.020], *P* < 0.001) exerted significant negative effects.

**Conclusions:**

Our findings demonstrate that ^18^F-FAPI-RGD PET/CT imaging is clinically applicable for CTD-ILD diagnosis. Future studies should explore its value in monitoring disease progression, treatment response assessment, and prognostic evaluation in CTD-ILD patients.

**Supplementary Information:**

The online version contains supplementary material available at 10.1186/s12931-025-03367-7.

## Introduction

Interstitial lung disease (ILD), known as diffuse parenchymal lung disease (DPLD), is a group of pulmonary diseases characterized by parenchymal inflammation and fibrosis, encompassing over 200 disease subtypes with significant heterogeneity. Connective tissue disease (CTD) is a major category of autoimmune diseases with inflammation of the blood vessels and connective tissues, which can easily lead to involvement of other vital organs and tissues. CTD-associated ILD (CTD-ILD) is the primary manifestation of lung involvement in CTD. Previous studies revealed that the incidence of ILD ranged from 1 to 31.5 per 100,000 person-years, with a prevalence between 6.3 and 71 per 100,000 person-years [1], while the overall incidence of CTD-ILD was reported to be 15% [2], which seriously affected the prognosis of patients. As an important tool, high resolution CT (HRCT) is a widely used for the diagnosis of CTD-ILD in clinical practice, but it is influenced by factors such as infections, heart failure, and artifacts. Additionally, patients who were classified as non-ILD based on HRCT may still experience varying degrees of impaired diffusing lung capacity for carbon monoxide (DLCO), and were at increased risk of ILD within the following 2 years [3]. These findings suggest that HRCT may not accurately identify CTD-ILD patients in certain cases, and diagnosis of ILD in CTD patients remains a challenge.

Lung fibrosis is a critical pathological characteristic of ILD and underlies the decline in lung function, acute exacerbations, and progression to progressive fibrosing ILD in patients with CTD-ILD [4, 5]. Although lung biopsy remains the gold standard for assessing interstitial fibrosis, its invasive nature and the limited scope of sampling restrict its clinical use.

Radioactive tracers, such as ^68^Ga/^18^F-labeled fibroblast activation protein inhibitors (FAPI), can specifically target fibroblast activation protein (FAP). Studies have shown increased uptake of FAPI tracers in reactive processes, fibrotic lesions, and inflammatory conditions. Notably, FAPI-based PET/CT tracers, such as ^68^Ga-FAPI-46, have been employed in the non-invasive detection of lung fibrosis in mouse models [6].

Integrins, a class of transmembrane glycoproteins, mediate cell-to-cell and cell-to-extracellular matrix adhesion and signal transduction. They regulate crucial cellular functions, including adhesion, migration, proliferation, and apoptosis, and are highly expressed on the surfaces of malignant tumor cells, endothelial cells in neovascularization, activated fibroblasts, macrophages, and osteoclasts. Arg-Gly-Asp(RGD) peptides, which bind strongly to αvβ3 and αvβ6 integrins, inhibit neovascular formation, making them promising agents for imaging and treating interstitial lung diseases characterized by early lung fibrosis and neovascularization [7].

The novel dual-target molecular probe ^18^F-FAPI-RGD simultaneously targets FAP and integrin receptors. ^68^Ga-FAPI-RGD PET/CT imaging has already been utilized in clinical imaging studies of various tumors [8]. This study aims to assess the feasibility and diagnostic efficacy of ^18^F-FAPI-RGD PET/CT for the diagnosis of ILD in CTD patients by analyzing the correlation between ^18^F-FAPI-RGD PET/CT imaging findings, lung function, inflammatory markers, and HRCT.

## Materials and methods

### Study population and criteria

This was a prospective observational study and was conducted between August 2023 and December 2024. Consecutively recruited patients first diagnosed with CTD-ILD at the ILD multidisciplinary outpatient clinic of Sichuan Provincial People’s Hospital, and CTD patients without ILD diagnosis during the same period were included as the control group. The study was approved by the Institutional Review Board of Sichuan Provincial People’s Hospital, University of Electronic Science and Technology of China (Process No. 2023 − 355) and carried out according to the principles of the Declaration of Helsinki. Informed consent was obtained from all participating patients.

The diagnostic criteria for CTD-ILD were based on the patient’s CTD history, combined with clinical symptoms and signs, lung imaging findings indicative of ILD (HRCT showing ground-glass opacities, consolidations, subpleural reticular opacities, honeycombing, traction bronchiectasis, etc.) according to the clinical guidelines [9, 10], and the exclusion of relevant factors such as pulmonary infectious diseases and cardiogenic diseases. Then a final diagnosis was made by a multidisciplinary team.

The diagnostic criteria for CTD subtypes followed their respective established diagnostic criteria, with definitive diagnosis confirmed by rheumatologists in the multidisciplinary clinic [11–16].

Exclusion criteria: (1) Other chronic lung diseases; (2) Pulmonary infectious diseases; (3) Cardiovascular diseases; (4) History of thoracic surgery; (5) Pregnancy and lactation; (6) Coexisting malignant tumors; (7) Refusal to participate; (8) Inability to cooperate for examinations.

### Data collection

General characteristics: age, gender, disease duration, smoking history, subtype of CTD, treatment strategies, pulmonary imaging features, and pulmonary function which contained forced expiratory volume in one second (FEV1), forced vital capacity (FVC), diffusing lung capacity for carbon monoxide (DLCO) and the percentage of their respective predicted values, such as FEV1 (%), FVC (%) and DLCO (%).

Laboratory data: white blood cells (WBC), neutrophils (NEU), lymphocytes (LYM), platelets (PLT), hypersensitive C-reactive protein (hsCRP), lactate dehydrogenase (LDH), erythrocyte sedimentation rate (ESR), ferritin (FER), interleukin-6 (IL-6) and Krebs Von den Lungen-6(KL-6). PET/CT and maximum standardized uptake value (SUVmax) of organs and tissues.

#### HRCT score

HRCT semi-quantitative visual scoring was independently performed by two experienced radiologists specializing in pulmonary imaging using established international grading criteria. The lungs were divided into six regions (bilateral upper, middle, and lower zones) at the levels of the carina and bilateral inferior pulmonary veins. Radiologists identified reticular patterns (characterized by interlobular or intralobular septal thickening) and honeycombing (clustered cystic airspaces with uniform diameter) based on Fleischner Society definitions [17]. The severity of reticular and honeycombing patterns was assessed per region using a Likert scale (0 = none; 1 = 1–25%; 2 = 26–50%; 3 = 51–75%; 4 = 76–100%) [18, 19], with the total lung involvement score representing the sum of all six regional scores (range: 0–24).

### Chemistry and radiochemistry

^18^F-Fluoride is produced by MINItrace cyclotron (GE Healthcare, USA). All FAPI-RGD (also denoted as LNC1007) precursors were provided by Yantai Lannacheng Biotechnology Co., Ltd. (Yantai, China). ^18^F-FAPI-RGD was radiolabeled manually. The detailed procedure for radiolabeling of FAPI-RGD variant was performed as described in previous publication [20]. The final product was diluted with saline and then passed through a microporous sterile filter. The radiochemical purity of ^18^F-FAPI-RGD is more than 95%.

### ^18^F-FAPI-RGD PET/CT protocol and procedures

Patients were not required to fast or control blood sugar levels before the examination. Following an intravenous injection of ^18^F-FAPI-RGD at a dose of approximately 4.81 MBq/kg, patients rested for 90–120 min before undergoing PET/CT scanning using the Biograph mCT Flow 64 (Siemens). The scan covered the area from the skull to either the upper thighs or the soles of the feet. The protocol began with non-enhanced CT acquisition (tube voltage: 120 keV; tube current: adjusted according to patient weight; slice thickness: 3 mm). PET images were acquired using continuous table motion technology. Post-scan, CT images were employed for attenuation correction of the PET images, and reconstruction was performed using the ordered-subset expectation-maximization algorithm. Lung data were reconstructed using specific algorithms designed for thin-layer lung imaging (slice thickness: 1.5 mm). Nuclear medicine physicians blinded to clinical and HRCT findings independently analyzed PET/CT images.

### Image interpretation and analysis

Bilateral lung SUVmax was obtained by scrolling through each transaxial slice and placing a 10-mm-diameter ROI over every visually identified area of increased 18 F-FDG uptake within the lung parenchyma; the single highest SUVmax value from both lungs was then recorded.

Two nuclear medicine physicians, each with over ten years of experience, independently analyzed the ^18^F-FAPI-RGD PET/CT images using the Syngo TrueD workstation (Siemens Medical Solutions). The analysis included both visual and semi-quantitative methods. PET, CT, and fused PET/CT images were compared frame-by-frame to visually assess the presence and distribution of interstitial lung diseases. For semi-quantitative analysis, regions of interest (ROIs) were drawn around areas of interstitial lung disease with high metabolic activity, and the maximum standardized uptake value was recorded. For different organs and tissues—such as the mediastinal blood pool, liver, spleen, left erector spinae, and subcutaneous fat—volumes of interest (VOIs) with a fixed diameter were selected and precisely positioned across various imaging planes (cross-sectional, sagittal, and coronal). The SUVmax values within the VOIs were automatically calculated and recorded.

### Sample size

To ensure the stability of the regression model and the reliability of results, sample size was calculated using Medcalc software based on anticipated AUC ≥ 0.8 (α = 0.05, power = 0.8). The final calculation shows that at least 45 patients are required.

### Statistical analysis

Continuous variables were analyzed using the Shapiro-Wilk test. Normally distributed data were expressed as mean ± standard deviation and calculated by Independent-samples T test. Non-normally distributed continuous data were presented as median [interquartile range] and assessed by the Mann-Whitney U test. Categorical data were showed as rates (percentages) and analyzed using the Chi-Square test for differences. Intergroup comparisons of continuous variables across multiple groups were performed using ANOVA or Kruskal-Wallis tests as appropriate, based on distributional characteristics (normality and homogeneity of variance). Associations between continuous clinical variables and SUVmax were evaluated using Pearson’s correlation analysis, whereas the relationship between the binary variable (CTD-ILD status) and continuous SUVmax values was assessed using point-biserial correlation. Parameters with statistically significant differences were subjected to multivariable logistic regression analysis, Prior to performing multivariable logistic regression analysis, considering that the original unit of the bilateral lung SUVmax might lead to poorly interpretable odds ratios (ORs) with excessively wide confidence intervals, we multiplied it by 10 to create a new variable(bilateral lung SUVmax (per 10 units)) for analysis. The final regression model included the transformed variable(bilateral lung SUVmax (per 10 units)). Receiver operating curve (ROC) was further generated for associated parameters. Subsequently, the area under the curve (AUC) was calculated, and the optimal cutoff value as well as sensitivity and specificity was measured using the Youden index. Multivariate linear analysis of parameters associated with SUVmax was performed. SPSS 26.0 was applied for statistical analysis, with *P* < 0.05 considered statistically significant.

## Results

### Grouping process

**Fig. 1:**
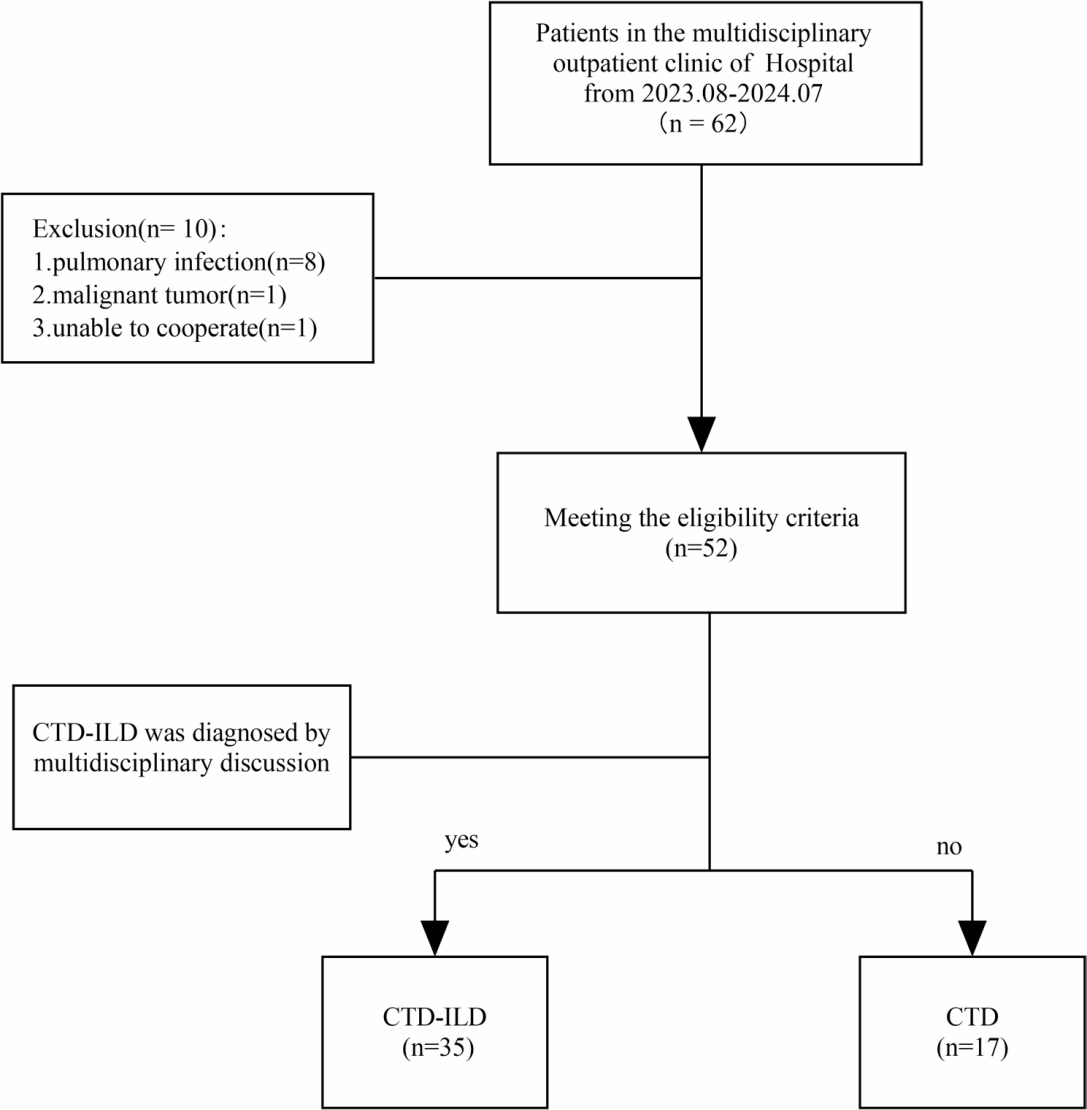
Flowchart of patient enrollment

A preliminary evaluation was conducted on all patients (*n* = 62) from the ILD multidisciplinary outpatient clinic up to December 2024. According to the exclusion criteria, 10 ineligible patients were excluded. Finally, 35 patients diagnosed with CTD, and 17 patients diagnosed with CTD-ILD were included. Clinical data from all enrolled patients were collected as planned, covering pulmonary function tests, HRCT score and ^18^F-FAPI-RGD PET/CT imaging (Fig. 1).

### General characteristics of patients

The average age of all patients was 56.9 years, with 20 males (38.5%) and 32 females (61.5%). The median disease duration was 12 months. 10 patients (19.2%) had smoking history. Among all patients, there were 28 cases (53.8%) of IIM, 9 cases (17.3%) of primary Sjogren syndrome, 5 case (9.6%) of systemic sclerosis, 7 cases (13.5%) of rheumatoid arthritis, and 3 cases (5.8%) of undifferentiated connective tissue disease (UCTD); pulmonary HRCT showed nonspecific interstitial pneumonia (NSIP) in 24 cases (68.6%), usual interstitial pneumonia (UIP) in 10 cases (28.6%), and organizing pneumonia (OP) in 1 case (2.8%).

The median IL-6 and KL-6 level of CTD-ILD patients was significantly higher than that of CTD patients (*P* < 0.05); The level of FEV_1_%, FVC%, and DLCO% were lower in the CTD-ILD group than in the CTD group (*P* < 0.001). There were no statistical differences in age, gender, smoking history, disease subtype, WBC, LYM, hsCRP, ESR, FER and treatment strategy between the two groups (Table 1).

**Table 1:** General characteristics of the enrolled patients

	Total	CTD-ILD	CTD	*P* ^#^
**N**	52	35	17	
Age(year)	56.9 ± 10.0	57.1 ± 9.2	56.7 ± 11.9	0.891
Gender (male)	20(38.5)	16(45.7)	4(23.5)	0.123
Smoking history (case)	10(19.2)	8(22.9)	2(11.8)	0.341
Disease subtype (case)				
IIM	28(53.8)	20(57.1)	8(47.1)	0.087
pSS	9(17.3)	4(11.4)	5(29.4)	
SSc	5(9.6)	5(14.3)	0(0)	
RA	7(13.5)	3(8.6)	4(23.5)	
UCTD	3(5.8)	3(8.6)	0(0)	
**Laboratory data**				
WBC(x10^9^/L)	7.8[6.0–8.7.0.7]	8.2[6.5–9.0.5.0]	7.0[5.6–8.1]	0.103
LYM(x10^9^/L)	1.4[0.9–1.9]	1.4[0.8–1.9]	1.3[1.0–2.0]	0.704
hsCRP(mg/L)	2.4[1.4–6.0.4.0]	2.7[1.7–6.9]	2.2[0.8–3.7]	0.110
ESR(mm/h)	40.0[23.0–63.0]	42.5[20.0–73.3.0.3]	34.0[24.0–53.5.0.5]	0.757
FER(ng/mL)	271.6[129.6–519.6.6.6]	373.5[187.8–731.7.8.7]	166.0[110.7–488.3.7.3]	0.086
IL-6(U/mL)	4.6[1.8–15.5]	5.7[3.1–26.5]	1.8[1.1–3.3]	0.016^*^
KL-6(U/ml)	595.4[386.0–1643.7.0.7]	1027.7[423.3–1906.5.3.5]	490.8[198.3–562.0]	0.010^*^
**HRCT feature** **(case)**				
NSIP	24(68.6)	24(68.6)	NA	NA
UIP	10(28.6)	10(28.6)	NA	
OP	1(2.8)	1(2.8)	NA	
**Pulmonary function**				
FEV_1_ (%)	86.4 ± 23.9	78.7 ± 21.1	102.4 ± 21.8	< 0.001^*^
FVC (%)	87.7[72.3–101.1.3.1]	76.4[65.4,90.3]	98.5[91.9,124.2]	< 0.001^*^
DLCO (%)	74.5[52.2,92.1]	56.2[50.2,75.2]	92.3[86.9,94.6]	< 0.001^*^
**Treatment strategy**				
GC	8(15.4)	6(17.1)	2(11.8)	0.684
IS	4(7.7)	2(5.7)	2(11.8)	
GC + IS	40(76.9)	27(77.1)	13(76.5)	

*IIM* idiopathic inflammatory myopathy, *pSS* primary Sjogren syndrome, *SSc* systemic sclerosis, *RA* rheumatoid arthritis *UCTD* undifferentiated connective tissue disease, *NSIP* nonspecific interstitial pneumonia, *UIP* usual interstitial pneumonia, *OP* organizing pneumonia, *WBC* white blood cell, *LYM* lymphocyte, *hsCRP* Hypersensitive C-reactive protein, *ESR* erythrocyte sedimentation rate, *FER* ferritin, *IL-6* interleukin-6, *KL-6* Krebs Von den Lungen-6, *FEV*_1_ forced expiratory volume in one second, *FVC* forced vital capacity, *DLCO* diffusing lung capacity for carbon monoxide, *GS* glucocorticoid, *IS* immunosuppressants

^#^The Comparison of differences between CTD-ILD and CTD

^*^P < 0.05

### Differences in ^18^F-FAPI-RGD PET/CT imaging and SUVmax

**Fig. 2:**
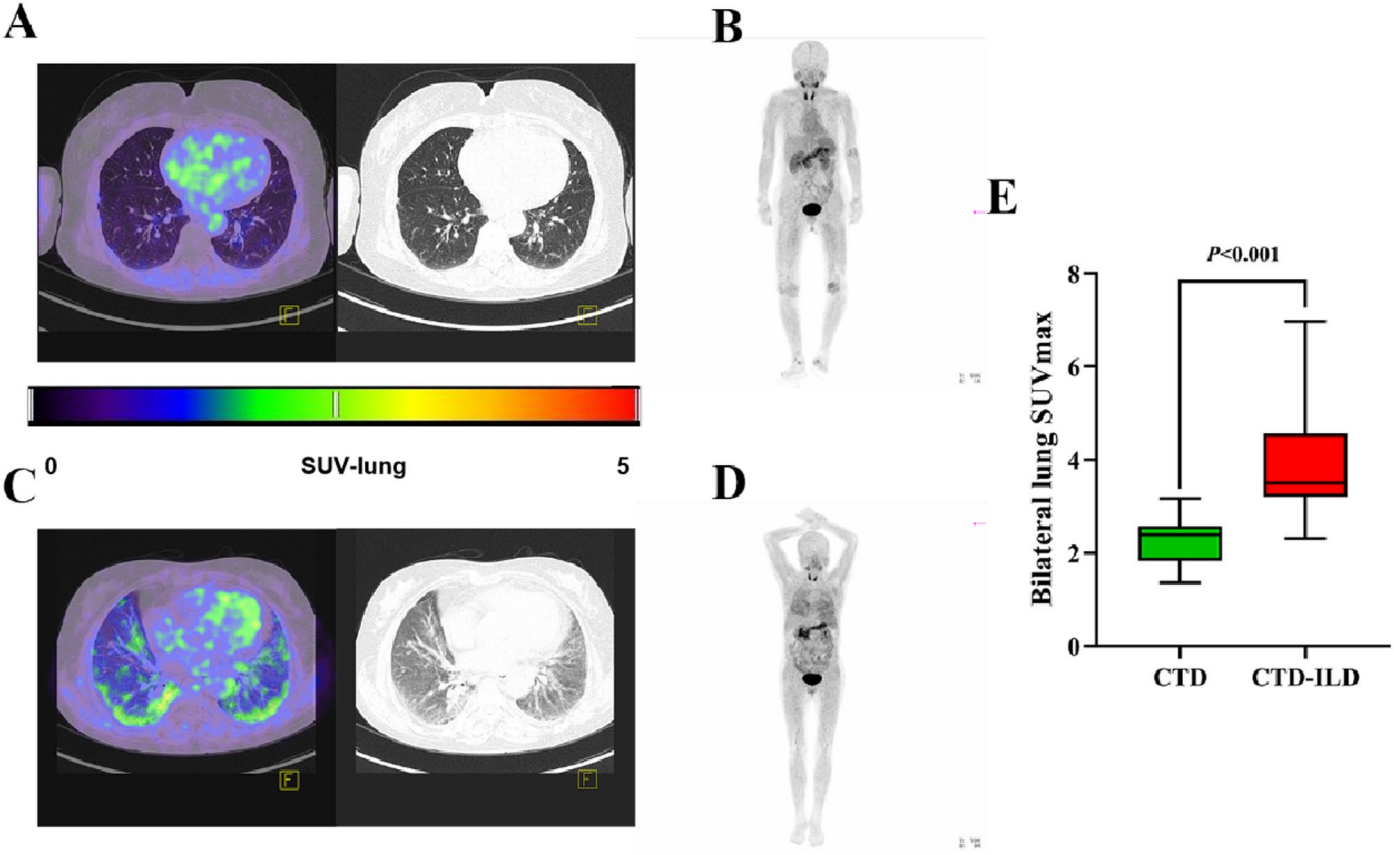
(**A**-**E**): Comparative analysis of pulmonary imaging features and bilateral lung SUVmax uptake between CTD and CTD-ILD patients via ^18^F-FAPI-RGD PET/CT. **A** and **B**: A 67-year-old female patient diagnosed with RA; **C** and **D**: A 62-year-old female patient diagnosed with dermatomyositis-associated interstitial lung disease; PET/CT: the corresponding transaxial PET emission scans and low-dose CT (**A** and **C**), together with the maximum intensity projection image of PET (**B** and **D**). Increased FAPI-RGD uptake in ILD patients (**C** and **D**) showed increased FAPI-RGD uptake in the areas of pulmonary fibrosis in bilateral lungs. **E**: The difference of bilateral lung SUVmax between CTD-ILD and CTD patients

**Fig. 3:**
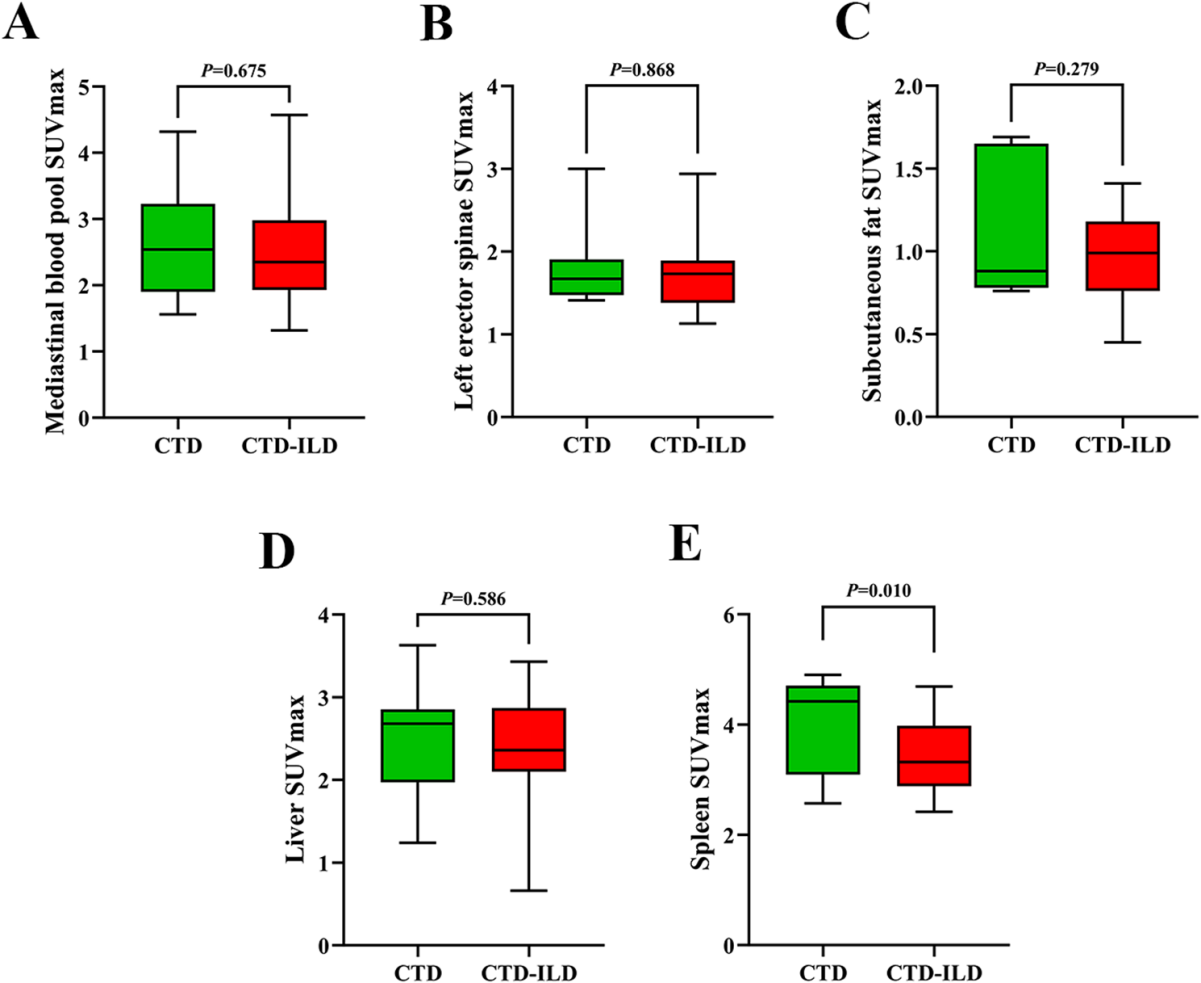
(**A**-**E**) Difference in SUVmax of organs and tissues(**A**: mediastinal blood pool, **B**: left erector spinae, **C**: subcutaneous fat, **D**: liver, **E**: spleen) between CTD and CTD-ILD

The results of ^18^F-FAPI-RGD PET/CT showed that the bilateral lung SUVmax in CTD-ILD patients increased significantly compared to CTD patients (*P* < 0.001) (Fig. 2), while spleen SUVmax in CTD-ILD patients decreased significantly compared to that in CTD patients(*P* = 0.010) (Fig. 3). No statistical differences were observed in the SUVmax of mediastinal blood pool, left erector spinae, subcutaneous fat and liver between two groups (Fig. 3).

Analysis across CTD-ILD subtypes demonstrated significantly elevated bilateral lung SUVmax in IIM-ILD(Supplementary Fig. 1), pSS-ILD(Supplementary Fig. 2), and RA-ILD(Supplementary Fig. 3) patients versus their non-ILD counterparts; concurrently, IIM-ILD(Supplementary Fig. 4) and pSS-ILD(Supplementary Fig. 5) patients exhibited significantly reduced spleen SUVmax compared to respective controls. RA-ILD patients showed significantly higher mediastinal blood pool SUVmax than controls (Supplementary Fig. 6). Representative ^18^F-FAPI-RGD PET/CT images and SUVmax values of organs/tissues from SSc-ILD and UCTD-ILD patients are presented in Supplementary Figs. 7 and Fig. 8 respectively, as disease-matched controls were unavailable for these subgroups. No statistically significant differences in bilateral lung SUVmax were observed among CTD and CTD-ILD subtypes (Supplementary Fig. 9). Analysis of distinct pulmonary HRCT patterns in CTD-ILD patients revealed no statistically significant differences in SUVmax of mediastinal blood pool, left erector spinae, subcutaneous fat, bilateral lung, liver and spleen between NSIP and UIP subtypes (Supplementary Fig. 10).

### Correlation between CTD-ILD and SUVmax values

Correlation analysis indicated that CTD-ILD was significantly positively correlated with bilateral lung SUVmax (*r* = 0.620, *P* < 0.001), spleen SUVmax (*r*=−0.361, *P* = 0.009) and subcutaneous fat SUVmax (*r*=−0.279, *P* = 0.045). However, CTD-ILD was not significantly correlated with SUVmax in the mediastinal blood pool, left erector spinae and liver (Table 2).

**Table 2:** Correlation analysis of SUVmax values of organs and tissues by PET/CT (^18^F-FAPI-RGD) in CTD-ILD patients

		bilateral lung	mediastinal blood pool	left erector spinae	liver	spleen	subcutaneous fat
CTD-ILD	r	0.620	−0.062	-0.033	−0.077	−0.361	−0.279
	*P*	< 0.001^*^	0.664	0.817	0.586	0.009^*^	0.045^*^

*CTD-ILD* connective tissue disease-associated interstitial lung disease

^*^P < 0.05

**Fig. 4:**
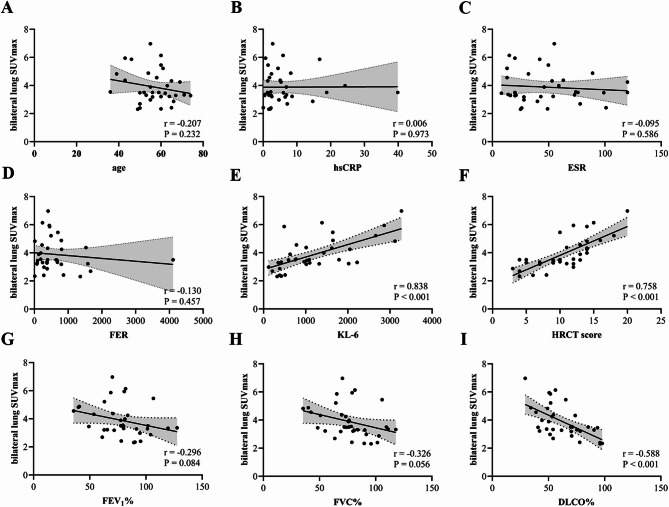
(**A**-**I**) Correlation analysis between bilateral lung SUVmax and clinical parameter in CTD-ILD patients(**A**: age, **B**: hsCRP, **C**: ESR, **D**: FER, **E**: KL-6; **F**: HRCT score; **G**: FEV1%; **H**: FVC%; **I**: DLCO%)

**Fig. 5:**
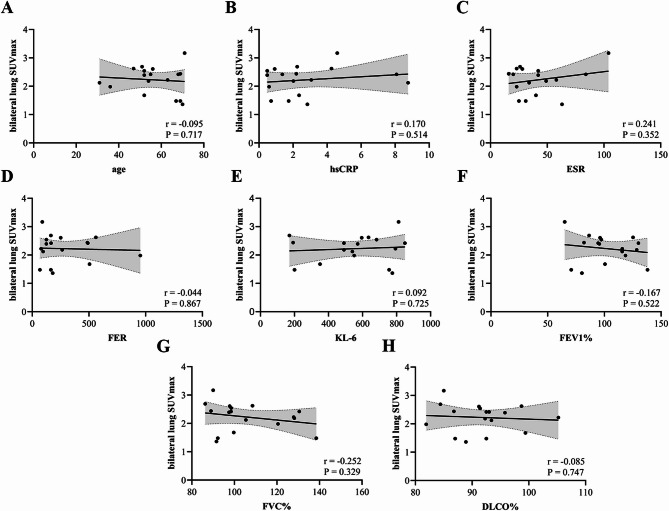
(A-H) Correlation analysis between bilateral lung SUVmax and clinical parameter in CTD patients(A: age, B: hsCRP, C: ESR, D: FER, E: KL-6; F: FEV1%; G: FVC%; H: DLCO%)

Correlation analysis revealed significant positive associations between bilateral lung SUVmax and both HRCT score (*r* = 0.758, *P* < 0.001) and KL-6 (*r* = 0.838, *P* < 0.001), while showing significant negative correlation with DLCO% (*r*=−0.588, *P* < 0.001) in CTD-ILD patients. No significant correlations were observed with age, hsCRP, ESR, FER, FEV₁%, or FVC% (Fig. 4). Conversely, in CTD patients, bilateral lung SUVmax demonstrated no statistically significant associations with age, hsCRP, ESR, FER, KL-6, FEV₁%, FVC%, or DLCO% (Figs. 5 ).

**Fig. 6:**
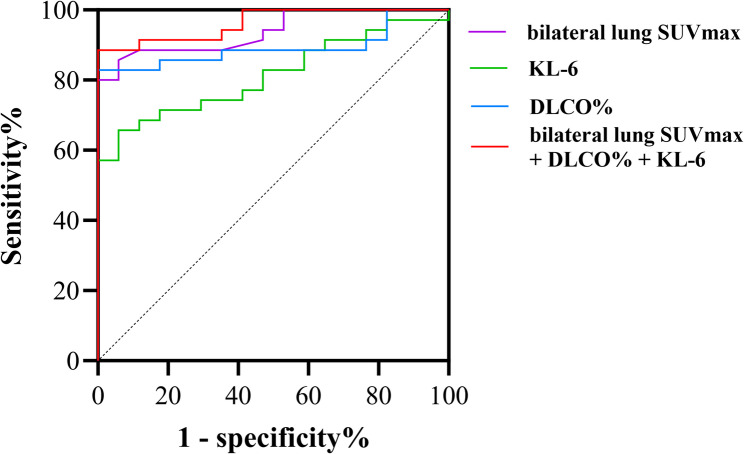
Diagnostic value of bilateral lung SUVmax, KL-6 and DLCO% in CTD-ILD

### Associated factors of CTD-ILD patients

Univariate Logistic regression showed that the KL-6(OR = 1.002, 95% CI= [1.000, 1.003], *P* = 0.021), DLCO%(OR = 0.860, 95% CI= [0.786, 0.942], *P* = 0.001), and bilateral lung SUVmax (per 10 units) (OR = 1.492, 95% CI= [1.177, 1.890], *P* = 0.001) were associated with CTD-ILD. Potential candidate variables were estimated by multivariate logistic regression analysis with the entry method. The results showed that bilateral lung SUVmax (per 10 units) (OR = 1.459, 95% CI= [1.031, 2.065], *P* = 0.033) was an independent associated factor for CTD-ILD after adjusting for age, KL-6 and DLCO% (Table 3).

**Table 3:** Logistic regression analysis of associated factors of CTD-ILD patients

	Univariate			Multivariate		
Variables	OR	95%CI	*P*	OR	95%CI	*P*
age	1.004	[0.947,1.064]	0.889	0.979	[0.874,1.098]	0.719
KL-6	1.002	[1.000,1.003]	0.021^*^	0.998	[0.995,1.002]	0.352
DLCO%	0.860	[0.786,0.942]	0.001^*^	0.932	[0.840,1.035]	0.189
bilateral lung SUVmax (per 10 units)	1.492	[1.177,1.890]	0.001^*^	1.459	[1.031,2.065]	0.033^*^

*OR* odds ratio, *CI* confidence interval, *KL-6* Krebs Von den Lungen-6, *DLCO* diffusing lung capacity for carbon monoxide

^*^P < 0.05

### Diagnostic Value of bilateral lung SUVmax in CTD-ILD patients

ROC curve analysis demonstrated that bilateral lung SUVmax yielded an AUC of 0.94 (95% CI [0.88, 1.00], *P* < 0.001) with an optimal cutoff of 3.18 (sensitivity 80.0%, specificity 100%); KL-6 showed an AUC of 0.81 (95% CI [0.70, 0.93], *P* < 0.001) at an optimal cutoff of 695.2 U/mL (sensitivity 65.7%, specificity 94.1%); DLCO% achieved an AUC of 0.89 (95% CI [0.80, 0.98], *P* < 0.001) with an optimal cutoff of 72.6% (sensitivity 88.2%, specificity 82.8%). The combined model incorporating these three markers produced an AUC of 0.96 (95% CI [0.92, 1.00], *P* < 0.001) with 88.6% sensitivity and 100% specificity (Fig. 6).

### Factors associated with bilateral lung SUVmax in CTD-ILD patients

Using multiple linear regression to assess the impact of age, KL-6, and DLCO% on bilateral lung SUVmax, we constructed a statistically significant model (F = 37.299, *P* < 0.001) where KL-6(β = 0.001 95%CI [< 0.001, 0.002], *P* < 0.001) and DLCO%(β=−0.031 95%CI [−0.043, −0.020], *P* < 0.001) collectively explained 68.1% of the variance in bilateral lung SUVmax (adjusted R²= 0.681). Age showed no significant association, with unstandardized regression coefficients and 95% CIs for each independent variable presented in the Table 4.

**Table 4:** Linear regression analysis of associated factors of bilateral lung SUVmax

Variables	β	95%CI	B	t	*P*	VIF	Tolerance
age	−0.012	[−0.032,1.064]	−0.092	−1.159	0.252	1.001	0.999
KL-6	0.001	[< 0.001, 0.002]	0.440	4.820	< 0.001^*^	1.333	0.750
DLCO%	−0.031	[−0.043,−0.020]	−0.516	−5.646	< 0.001^*^	1.334	0.749

Note: F = 37.299, *P* < 0.001; adjusted R²= 0.681; Durbin-Watson statistic = 2.37

*β* unstandardized regression coefficient, *CI* confidence interval, *B* standardized partial regression coefficient, *VIF* variance inflation factor, *KL-6* Krebs Von den Lungen-6, *DLCO* diffusing lung capacity for carbon monoxide

^*^P < 0.05

## Discussion

Interstitial lung disease (ILD) encompasses a broad spectrum of highly heterogeneous pulmonary disorders, where early detection has emerged as a critical research focus. PET/CT imaging with targeted tracers represents a non-invasive approach offering significant value for individualized early diagnosis of ILD patients [21]. As a prevalent ILD subtype, our study systematically evaluated the diagnostic potential of ¹⁸F-FAPI-RGD PET/CT in clinical CTD-ILD assessment for the first time, laying a foundation for future research in this field.

Using ¹⁸F-FAPI-RGD PET/CT imaging, we demonstrated significantly elevated lung SUVmax in CTD-ILD patients with strong predictive value for ILD. PET/CT technology enables non-invasive assessment of cellular metabolism and high-resolution imaging of pulmonary parenchymal structures. Within this domain, ¹⁸F-FDG and ⁶⁸Ga-FAPI represent key tracers. While Groves et al. observed increased ¹⁸F-FDG uptake in ILD lungs [22]—suggesting potential for early detection [23]—its elevated retention in pulmonary infections [24] complicates differentiation from infectious pathologies. Our ¹⁸F-FAPI-RGD approach specifically targets localized fibroblasts, activated myofibroblasts, and fibrotic maturation status in pulmonary lesions. This dual-pathway assessment offers distinct advantages: FAPI outperforms FDG in discriminating infectious processes [25], while RGD-bound integrin αvβ3 exhibits consistently elevated expression across all ILD subtypes [26]. Given scarce research on PET/CT tracers for early ILD diagnosis and absence of dual-target probes in this domain, our findings confirm ¹⁸F-FAPI-RGD’s diagnostic efficacy for CTD-ILD.

In connective-tissue-disease-associated interstitial lung disease (CTD-ILD), research on FAP-targeted imaging is still in its infancy; nevertheless, several early studies have already highlighted its clinical potential—findings that parallel our own. In a comprehensive cohort of various ILDs, Yang et al. [27] first demonstrated that FAP expression is markedly up-regulated at the earliest stages of pulmonary fibroblast activation and correlates closely with the extent of fibrotic foci. Subtype-specific investigations provide further support: in systemic sclerosis–associated ILD (SSc-ILD), Bergmann et al. [28] reported that both mean (SUVmean) and maximum (SUVmax) ⁶⁸Ga-FAPI-04 PET-CT uptake were significantly higher than in controls and strongly associated with disease activity and progression risk. Similarly, in a cohort of idiopathic inflammatory myopathy–associated ILD (IIM-ILD) [29], the target-to-background ratios derived from pulmonary SUVmax versus mediastinal blood pool (TBRmax and TBRmean) were markedly elevated compared with non-ILD and healthy controls, and correlated significantly with HR-CT lesion extent, NYHA class, and declining pulmonary function—underscoring the potential of FAPI PET for assessing active fibrosis and risk stratification in CTD-ILD. Our tracer incorporates FAP within a dual-target construct; consistent with these findings, we observed significantly higher pulmonary SUVmax in ILD patients than in non-ILD controls, providing real-time evidence of ongoing fibrotic activity across the whole lung in CTD-ILD.

Our study revealed significantly reduced spleen SUVmax in ILD patients versus controls, contrasting with elevated bilateral lung SUVmax. Given RGD’s specific binding to multiple integrin subtypes within the tracer, we postulate this may relate to differential site-specific integrin activation mediating distinct physiological processes. Prior studies demonstrate that adhesion molecules facilitate inflammatory cell recruitment to lungs [30], potentially contributing to ILD pathogenesis. Further evidence indicates integrins promote macrophage migration to pulmonary tissue, driving inflammation [31], while activated integrins enhance monocyte recruitment and collagenase activity—culminating in inflammatory tissue damage [32]. Consequently, in CTD-ILD patients, whether splenic monocytes and macrophages migrate to lungs (promoting pulmonary inflammation and fibrosis) warrants dedicated investigation.

Further exploration of factors associated with bilateral lung SUVmax revealed a significant positive correlation with KL-6 levels. As a chemokine secreted by proliferating or damaged type II alveolar epithelial cells, elevated KL-6 promotes pulmonary fibroblast proliferation [33, 34].Concurrently, type II epithelial cell injury induces integrin αvβ6 expression [35], activating TGF-β signaling to drive fibrogenesis [36]. Conversely, DLCO% showed significant negative correlation with bilateral lung SUVmax. The core mechanism involves destruction of the alveolar-capillary membrane architecture, impairing diffusion capacity. Greater type II epithelial damage in ILD corresponds to lower DLCO values [37]. Collectively, these findings establish bilateral lung SUVmax as a robust biomarker of pulmonary tissue damage and fibrotic progression in ILD.

Among the subjects we included in the study, there were no significant differences between CTD-ILD patients and pure CTD patients in terms of age, gender, smoking history, and disease subtype, which helps to minimize confounding factors in the subsequent pulmonary imaging analysis. Meanwhile, our study showed that compared with the CTD control group, CTD-ILD patients had significantly reduced lung function (FEV1%, FVC%, and DLCO%), significantly increased serum IL-6 levels, and a significant increase in KL-6, which is closely related to alveolar and capillary damage, activation of immune and inflammatory status, and fibrotic activity in ILD patients as mentioned in the existing literature [38–41].

Our study has several limitations. First, the small sample size of this pilot study necessitates larger studies to further validate our findings. Second, the patients in this study had varying disease durations and activity, making it unclear whether these factors influenced the imaging results. Third, while this study focused on the diagnostic value of ^18^F-FAPI-RGD PET/CT for ILD, future research should also investigate disease progression, efficacy evaluation, and prognostic analysis. Finally, our study cannot conclusively establish whether reduced spleen SUVmax in CTD-ILD may reflect macrophage or monocyte migration from spleen to lungs, warranting further mechanistic exploration. Beyond assessing lung involvement, it is worth exploring the use of ^18^F-FAPI-RGD PET/CT for diagnosing and predicting disease activity and prognosis in other organs and tissues affected by rheumatic and immunological diseases.

## Conclusions

The results from using ^18^F-FAPI-RGD PET/CT imaging technology showed that bilateral lung SUVmax in CTD-ILD patients was significantly higher than in non-ILD patients, demonstrating a strong correlation with ILD in CTD and high diagnostic value. Future studies should expand the sample size and explore disease progression, efficacy evaluation, and prognosis analysis in a more comprehensive, multi-dimensional manner.

## Supplementary Information

Supplementary Material 1.

## Data Availability

Data are available from the corresponding author on reasonable request.
