# Supplementary material for: Pilot study of 18F-FAPI-RGD PET/CT for the diagnosis of connective tissue disease associated interstitial lung disease
Source: Respir Res. 2025 Nov 15;26:317. doi: 10.1186/s12931-025-03367-7 (PMC12619180; doi:10.1186/s12931-025-03367-7)
Supplement: Supplementary file 1 — Supplementary Material 1. [file 12931_2025_3367_MOESM1_ESM.docx]

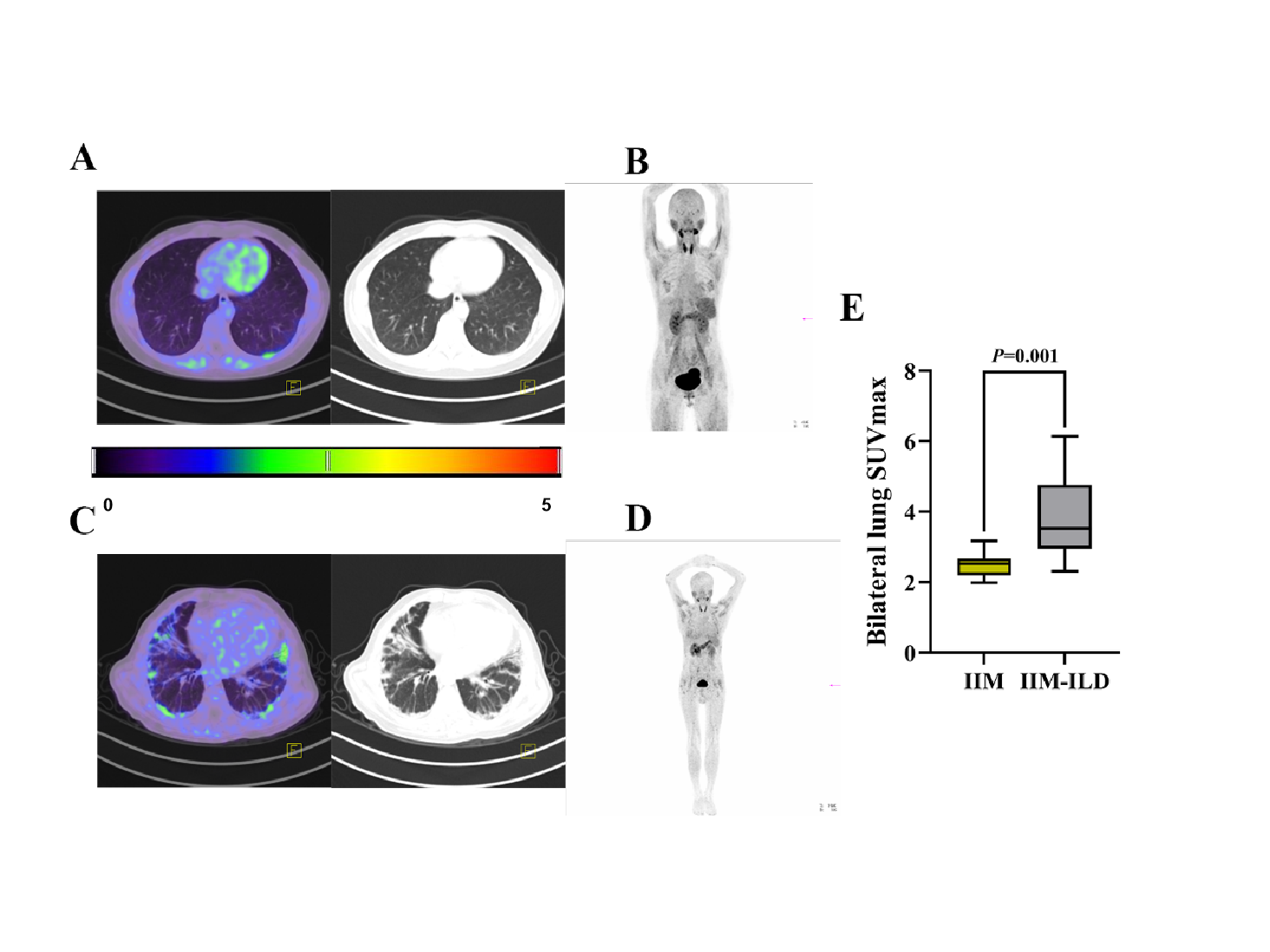


Supplementary Figure 1(A-E): Comparative analysis of pulmonary imaging features and bilateral lung SUVmax uptake between IIM and IIM-ILD patients via ^18^F-FAPI-RGD PET/CT.

A and B: A 31-year-old female patient diagnosed with anti-synthetase syndromea ssociated interstitial lung disease; C and D: A 64-year-old male patient diagnosed with dermatomyositis-associated interstitial lung disease;

PET/CT: the corresponding transaxial PET emission scans and low-dose CT (A and C), together with the maximum intensity projection image of PET (B and D). Increased FAPI-RGD uptake in ILD patients (C and D) showed increased FAPI-RGD uptake in the areas of pulmonary fibrosis in bilateral lungs. E: The difference of bilateral lung SUVmax between IIM and IIM-ILD patients.


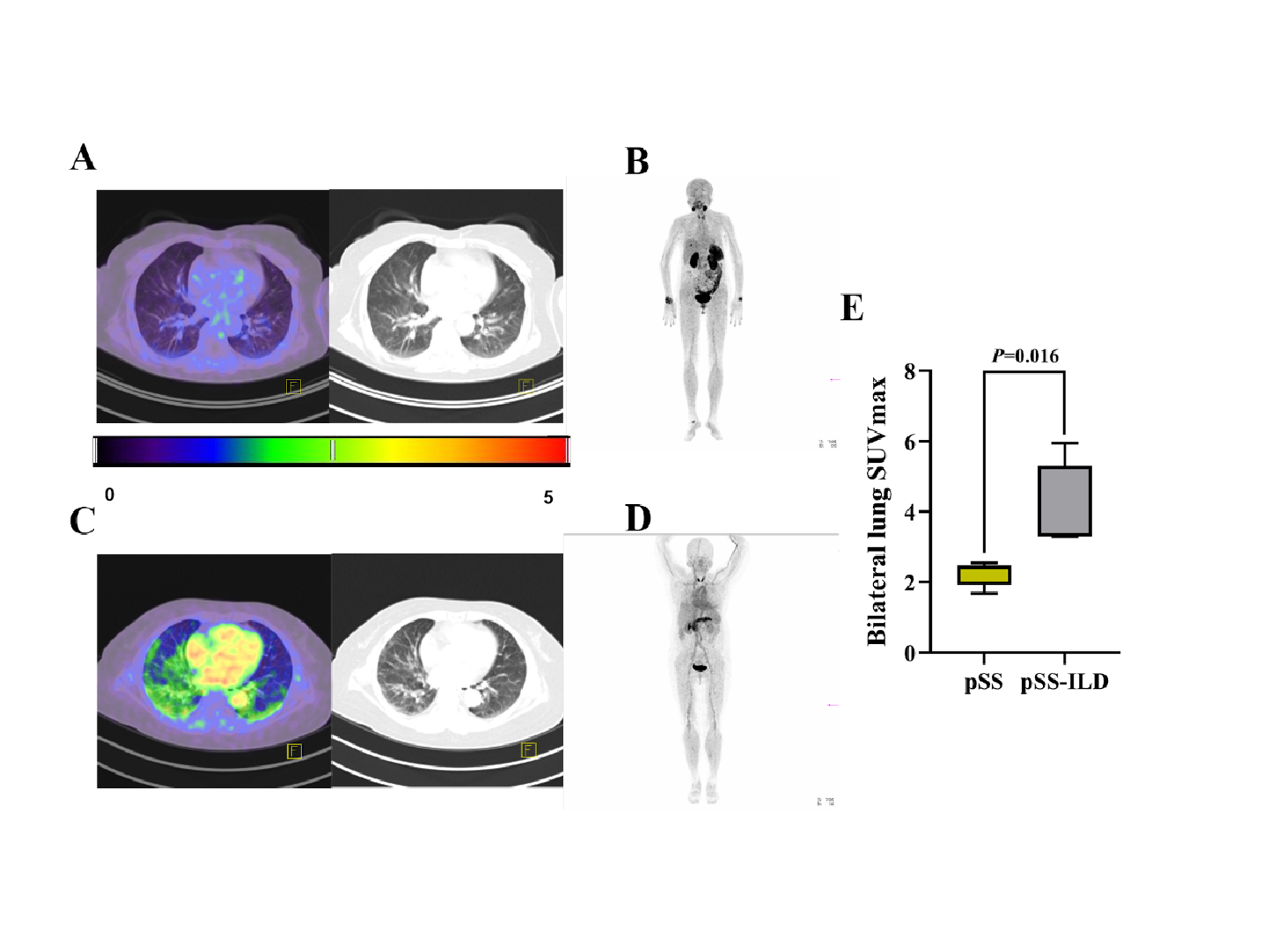


Supplementary Figure 2(A-E): Comparative analysis of pulmonary imaging features and bilateral lung SUVmax uptake between pSS and pSS-ILD patients via ^18^F-FAPI-RGD PET/CT.

A and B: A 55-year-old female patient diagnosed with pSS; C and D: A 71-year-old female patient diagnosed with pSS-ILD;

PET/CT: the corresponding transaxial PET emission scans and low-dose CT (A and C), together with the maximum intensity projection image of PET (B and D). Increased FAPI-RGD uptake in ILD patients (C and D) showed increased FAPI-RGD uptake in the areas of pulmonary fibrosis in bilateral lungs. E: The difference of bilateral lung SUVmax between pSS and pSS-ILD patients.


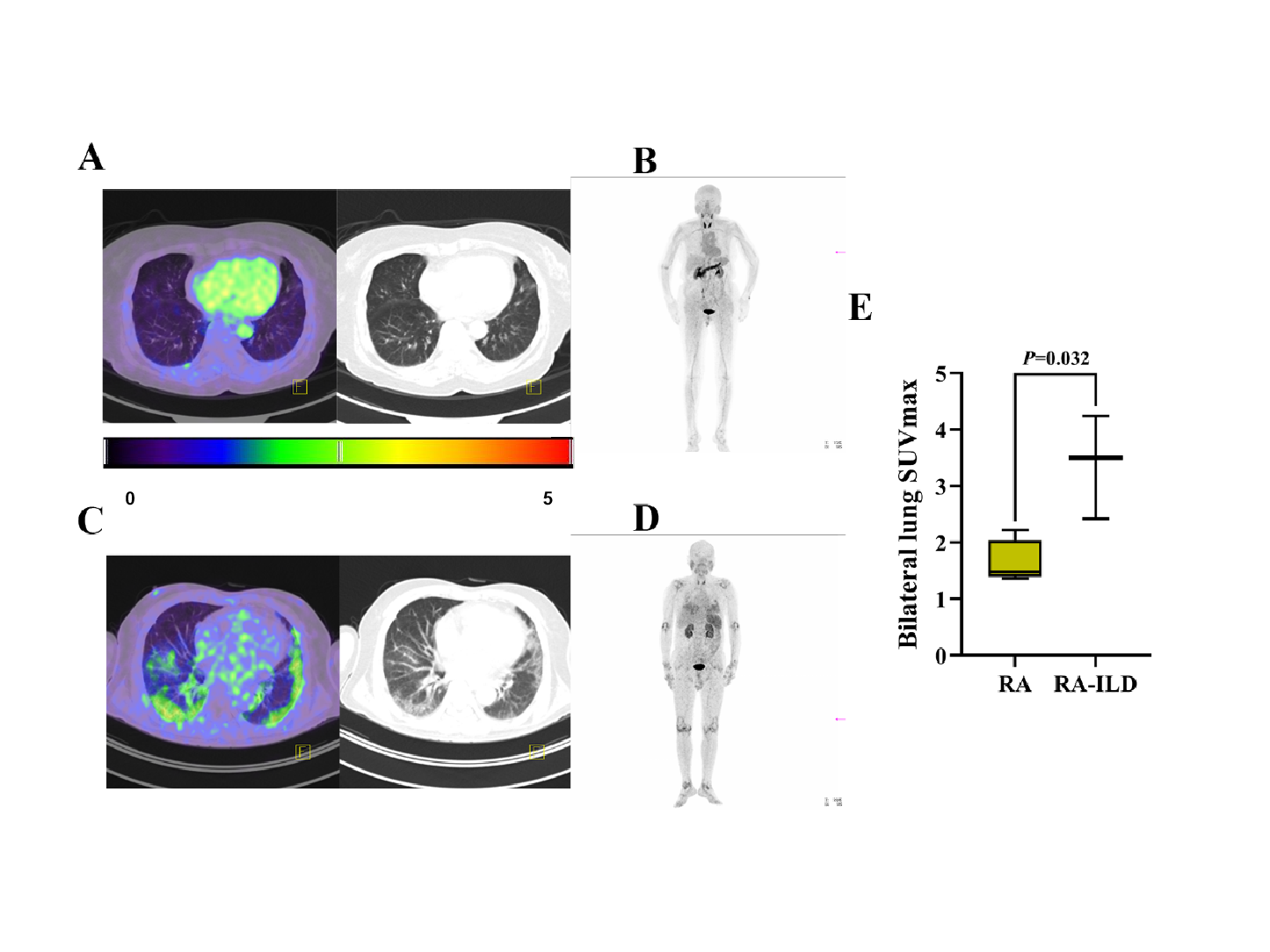


Supplementary Figure 3(A-E): Comparative analysis of pulmonary imaging features and bilateral lung SUVmax uptake between RA and RA-ILD patients via ^18^F-FAPI-RGD PET/CT.

A and B: A 69-year-old female patient diagnosed with RA; C and D: A 69-year-old male patient diagnosed with RA-ILD;

PET/CT: the corresponding transaxial PET emission scans and low-dose CT (A and C), together with the maximum intensity projection image of PET (B and D). Increased FAPI-RGD uptake in ILD patients (C and D) showed increased FAPI-RGD uptake in the areas of pulmonary fibrosis in bilateral lungs. E: The difference of bilateral lung SUVmax between RA and RA-ILD patients.


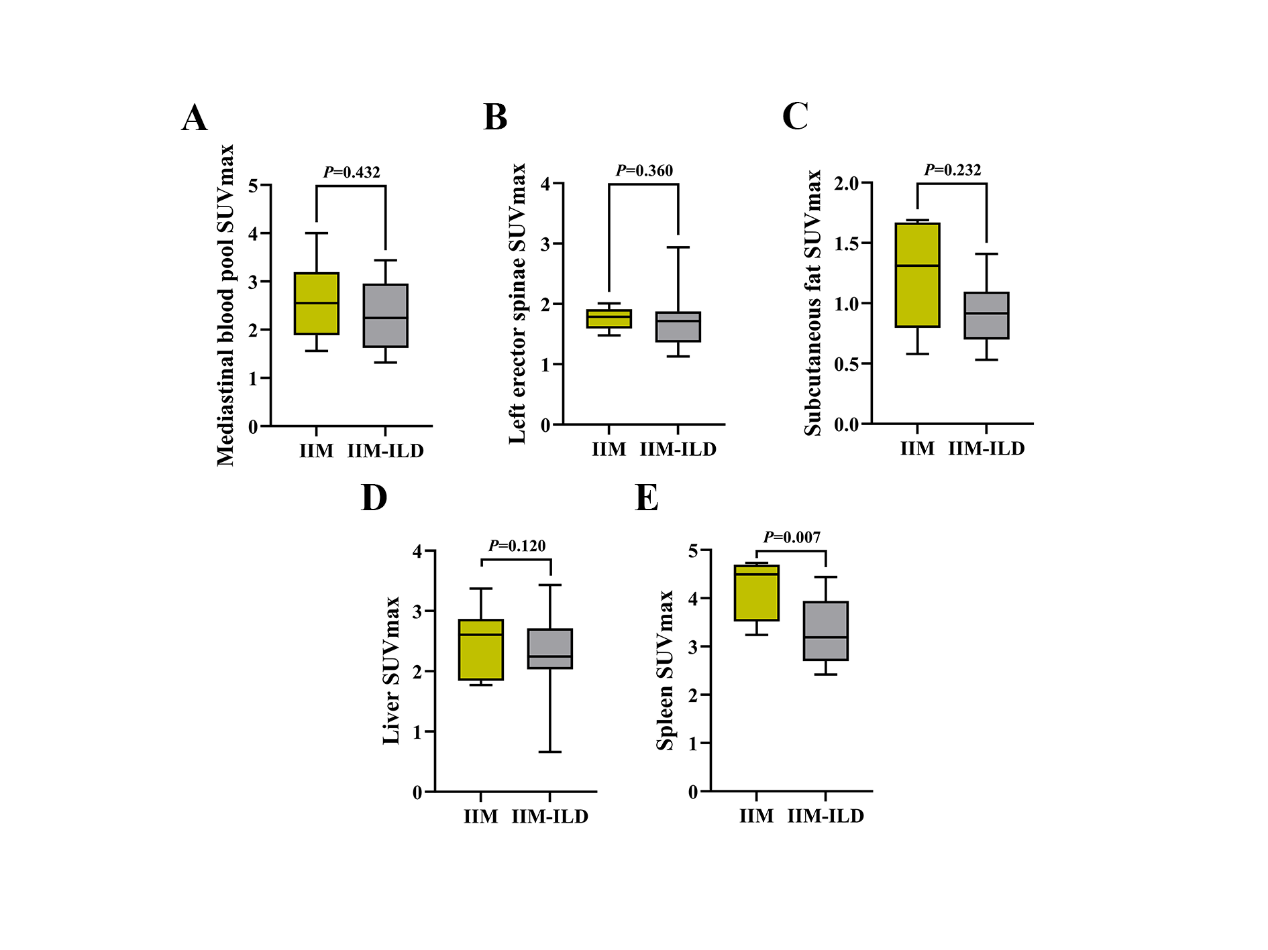


Supplementary Figure 4(A-E) Difference in SUVmax of organs and tissues between IIM and IIM-ILD.

(A: mediastinal blood pool, B: left erector spinae, C: subcutaneous fat, D: liver, E: spleen)


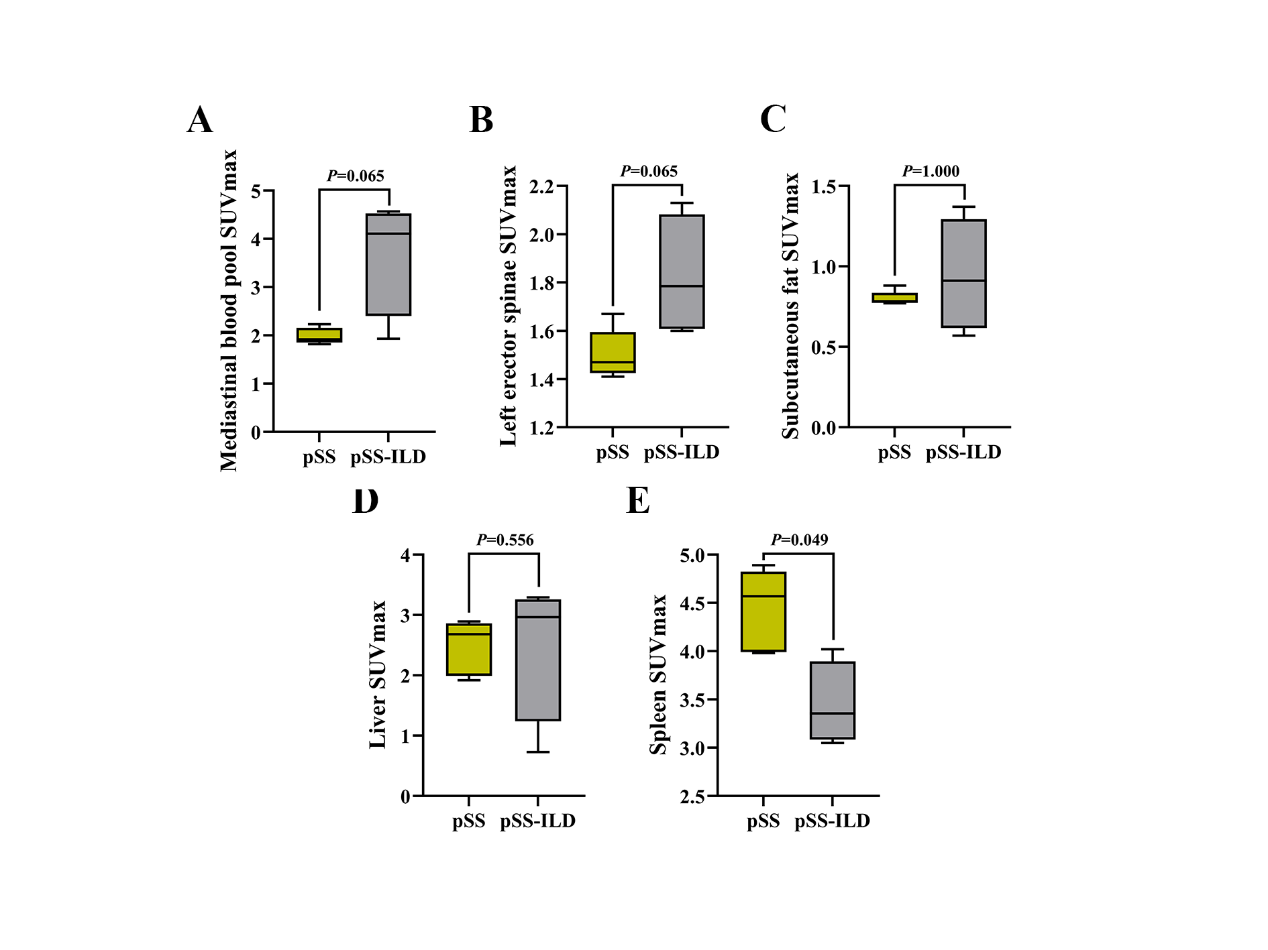


Supplementary Figure 5(A-E) Difference in SUVmax of organs and tissues between pSS and pSS-ILD.

(A: mediastinal blood pool, B: left erector spinae, C: subcutaneous fat, D: liver, E: spleen)


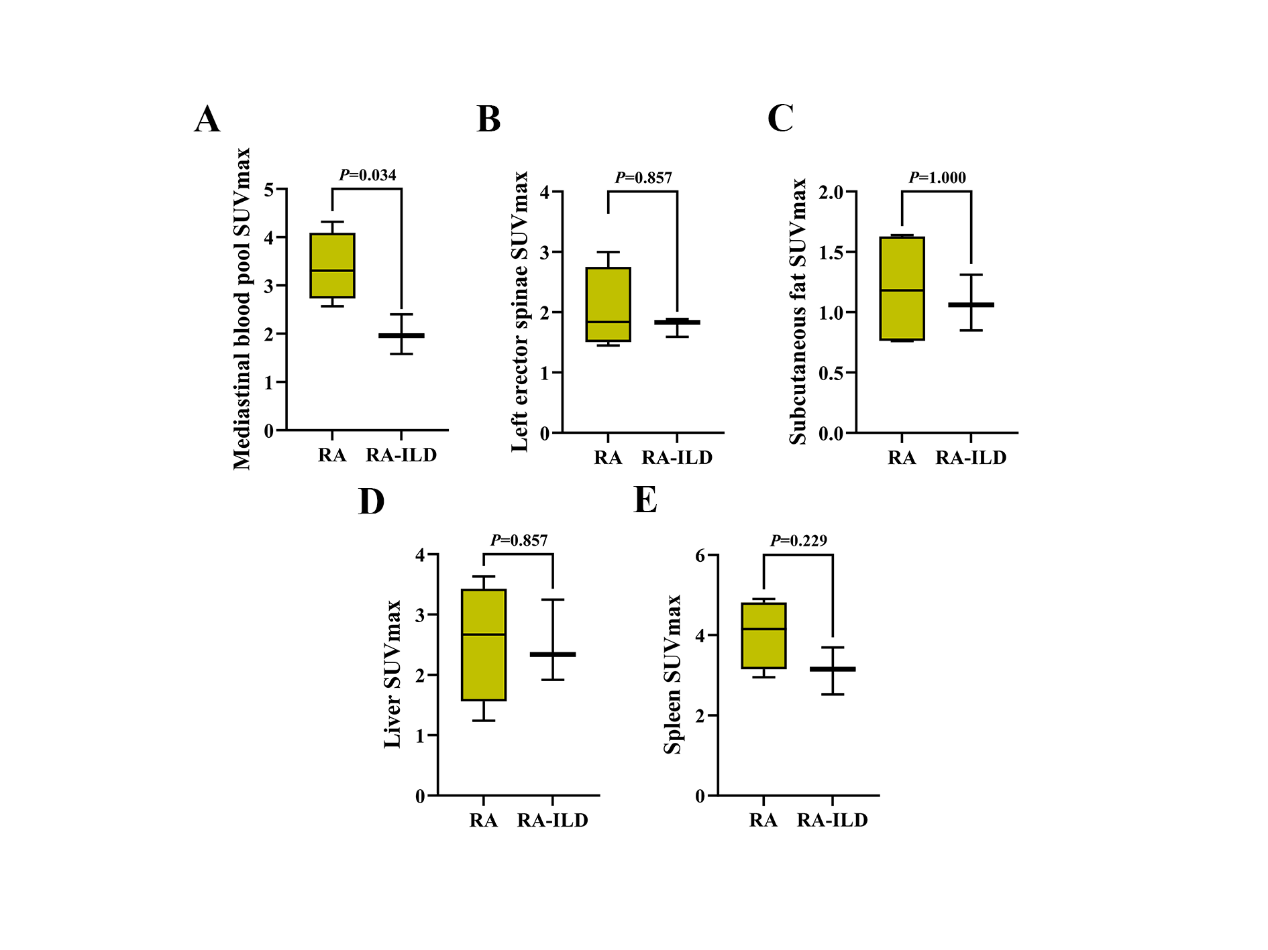


Supplementary Figure 6(A-E) Difference in SUVmax of organs and tissues between RA and RA-ILD.

(A: mediastinal blood pool, B: left erector spinae, C: subcutaneous fat, D: liver, E: spleen)


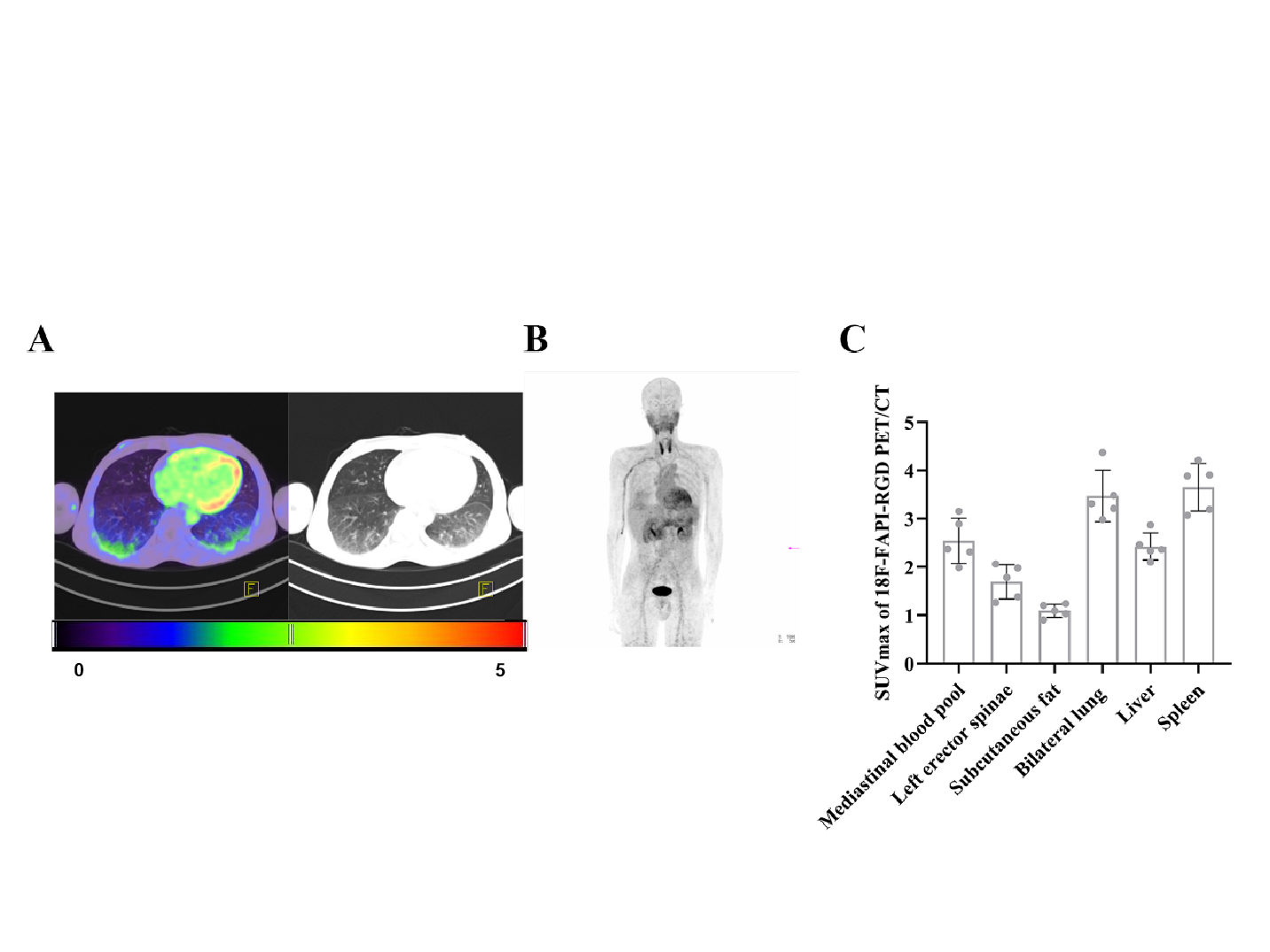


Supplementary Figure 7(A-B): Pulmonary imaging features and SUVmax of organs and tissues uptake in SSc-ILD patients via ^18^F-FAPI-RGD PET/CT.

A and B: A 55-year-old male patient diagnosed with SSc-ILD;

PET/CT: the corresponding transaxial PET emission scans and low-dose CT (A), together with the maximum intensity projection image of PET (B) showed increased FAPI-RGD uptake in the areas of pulmonary fibrosis in bilateral lungs. C: The SUVmax of organs and tissues in SSc-ILD patients.


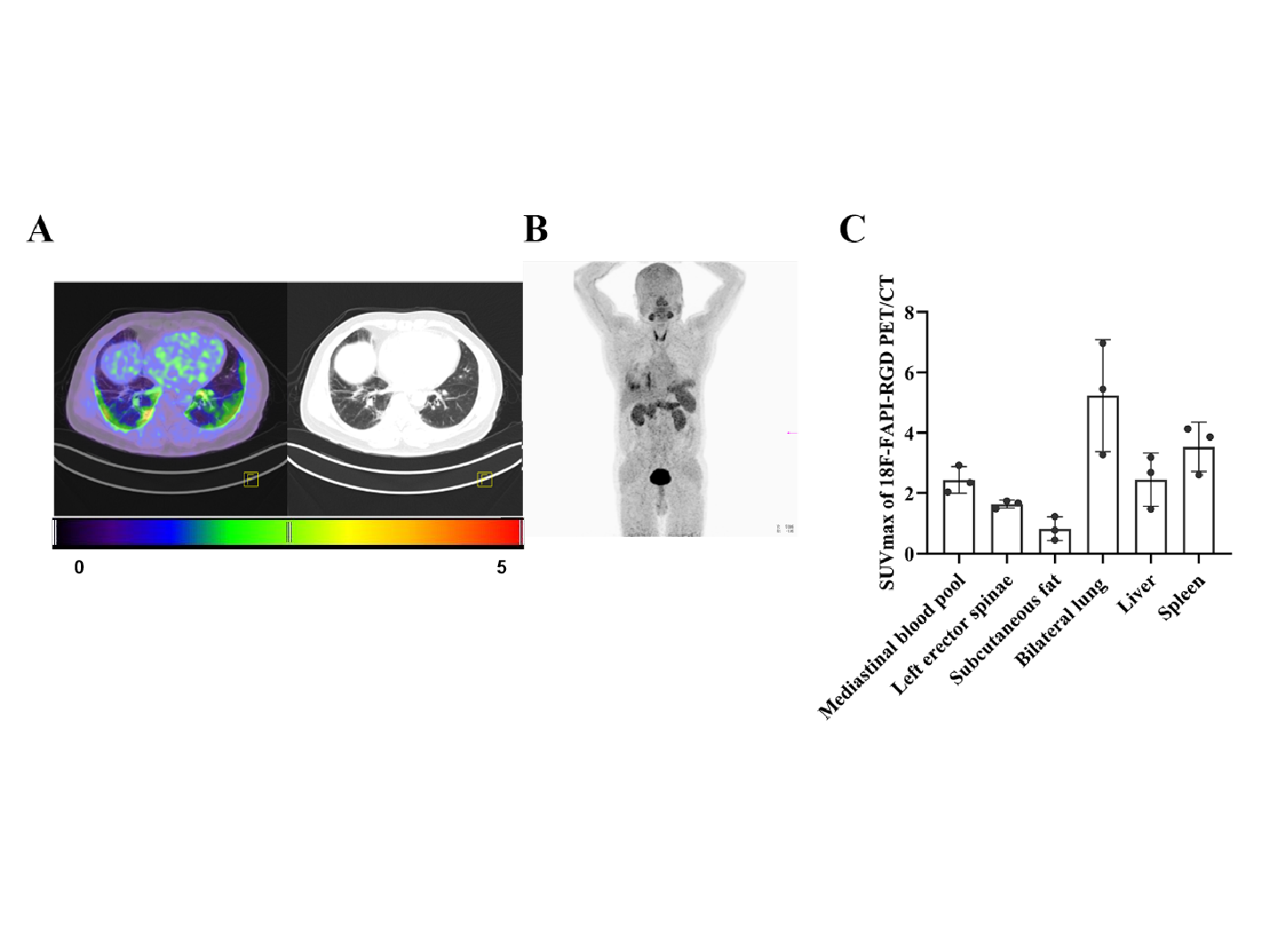


Supplementary Figure 8(A-B): Pulmonary imaging features and SUVmax of organs and tissues uptake in UCTD-ILD patients via ^18^F-FAPI-RGD PET/CT.

A and B: A 60-year-old male patient diagnosed with UCTD-ILD;

PET/CT: the corresponding transaxial PET emission scans and low-dose CT (A), together with the maximum intensity projection image of PET (B) showed increased FAPI-RGD uptake in the areas of pulmonary fibrosis in bilateral lungs. C: The SUVmax of organs and tissues in UCTD-ILD patients.


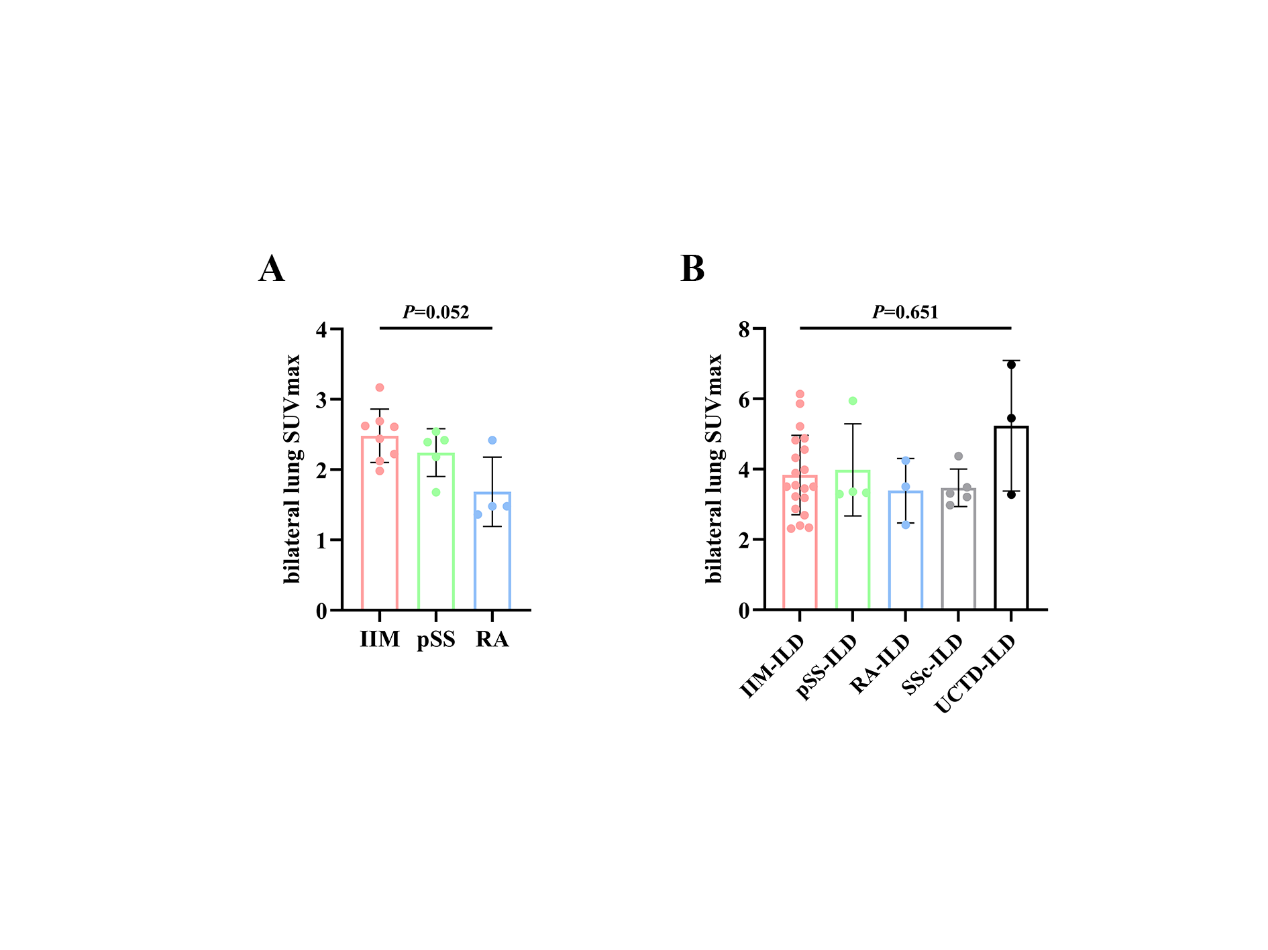


Supplementary Figure 9(A-B) Comparative analysis of bilateral lung SUVmax uptake among CTD and CTD-ILD subgroups.

(A:CTD subgroup; B: CTD-ILD subgroup)


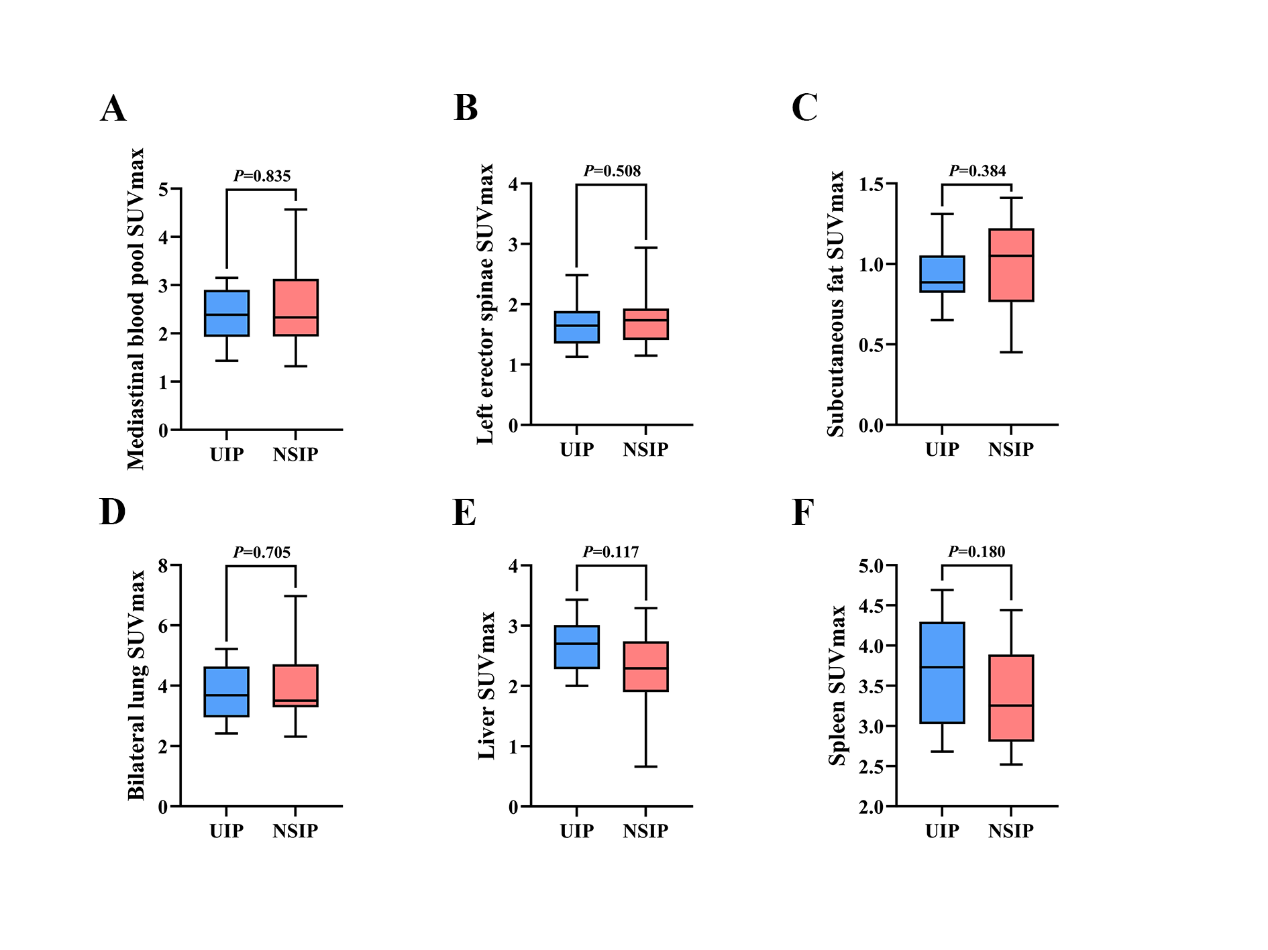


Supplementary Figure 10(A-F) Difference in SUVmax of organs and tissues between UIP and NSIP in CTD-ILD patients.

(A: mediastinal blood pool; B: left erector spinae; C: subcutaneous fat; D: bilateral lung; E: liver; F: spleen)
